# Progress in the correlation of postoperative cognitive dysfunction and Alzheimer's disease and the potential therapeutic drug exploration

**DOI:** 10.1002/ibra.12040

**Published:** 2022-05-19

**Authors:** Dong‐Qin Chen, Xu Fang, Zhao‐Qiong Zhu

**Affiliations:** ^1^ Department of Anesthesiology Affiliated Hospital of Zunyi Medical University Zunyi China; ^2^ College of Anesthesiology Zunyi Medical University Zunyi China

**Keywords:** Alzheimer's disease, medical treatment, pathogenesis, postoperative cognitive dysfunction

## Abstract

Postoperative cognitive dysfunction (POCD) is a decrease in mental capacity that can occur days to weeks after a medical procedure and may become permanent and rarely lasts for a longer period of time. With the continuous development of research, various viewpoints in academic circles have undergone subtle changes, and the role of anesthesia depth and anesthesia type seems to be gradually weakened; Alzheimer's disease (AD) is a latent and progressive neurodegenerative disease in the elderly. The protein hypothesis and the synaptic hypothesis are well‐known reasons. These changes will also lead to the occurrence of an inflammatory cascade. The exact etiology and pathogenesis need to be studied. The reasonable biological mechanism affecting brain protein deposition, neuroinflammation, and acetylcholine‐like effect has a certain relationship between AD and POCD. Whereas there is still further uncertainty about the mechanism and treatment, and it is elusive whether POCD is a link in the continuous progress of AD or a separate entity, which has doubts about the diagnosis and treatment of the disease. Therefore, this review is based on the current common clinical characteristics of AD and POCD, and pathophysiological research, to search for their common points and explore the direction and new strategies for future treatment.

## Graphical abstract

There is a correlation between postoperative cognitivedysfunction and Alzheimer's disease in clinical symptoms, cerebrospinal fluid markers, and the expression of some inflammatory factors, and in mechanisms, the deposition of A‐β and tau proteins, and cholinergic system disorders, synaptic plasticity plays an important role in addressing these commonalities, anti‐inflammatory, clearing A‐β and tau proteins, maintaining the balance of the intestinal flora, exosomes, and even microRNAs are potential therapeutic targets.

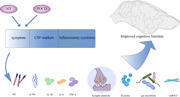

## 1 INTRODUCTION

**Figure 1:**
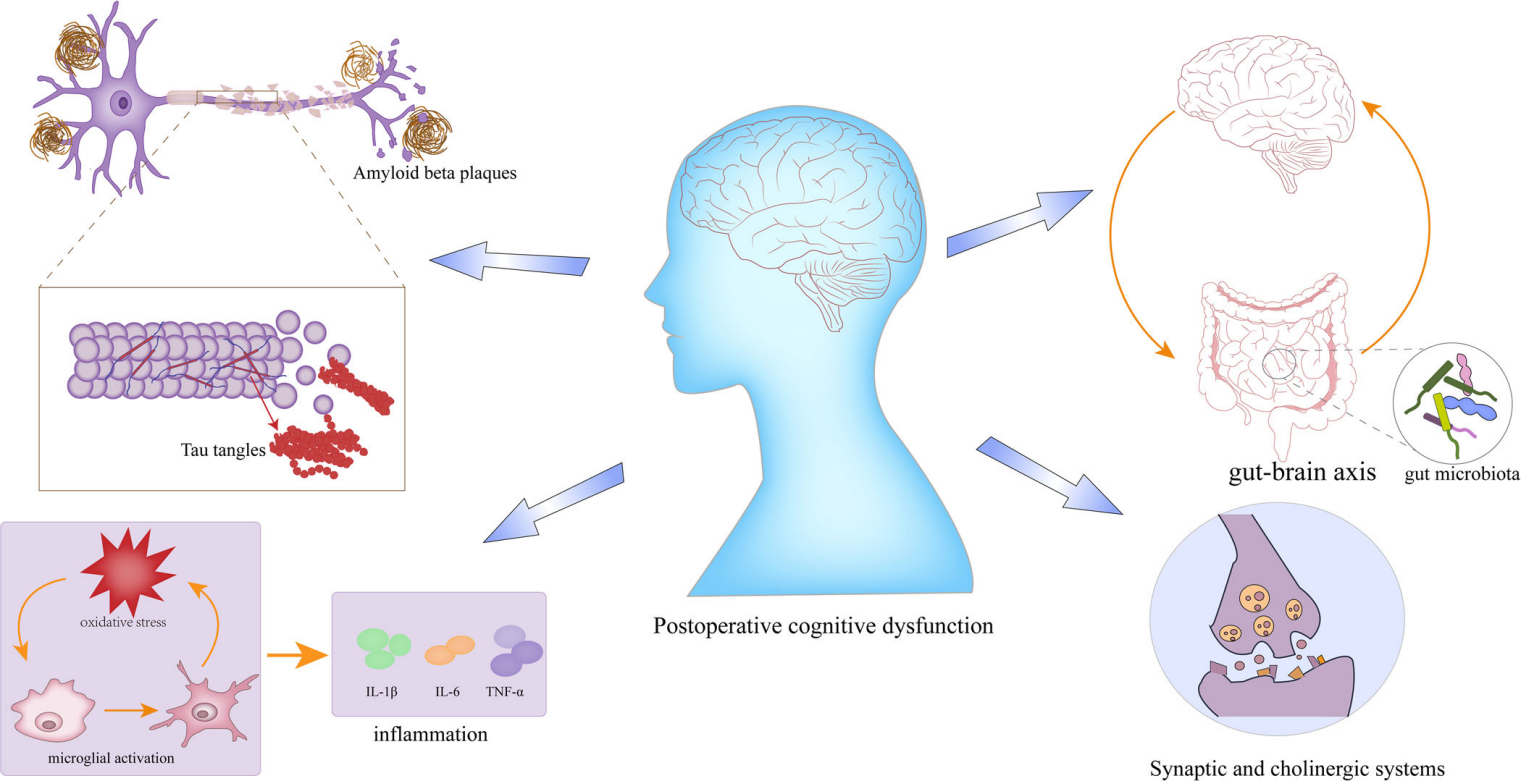
Possible pathogenesis and target of postoperative cognitive dysfunction. [Color figure can be viewed at wileyonlinelibrary.com]

**Figure 2:**
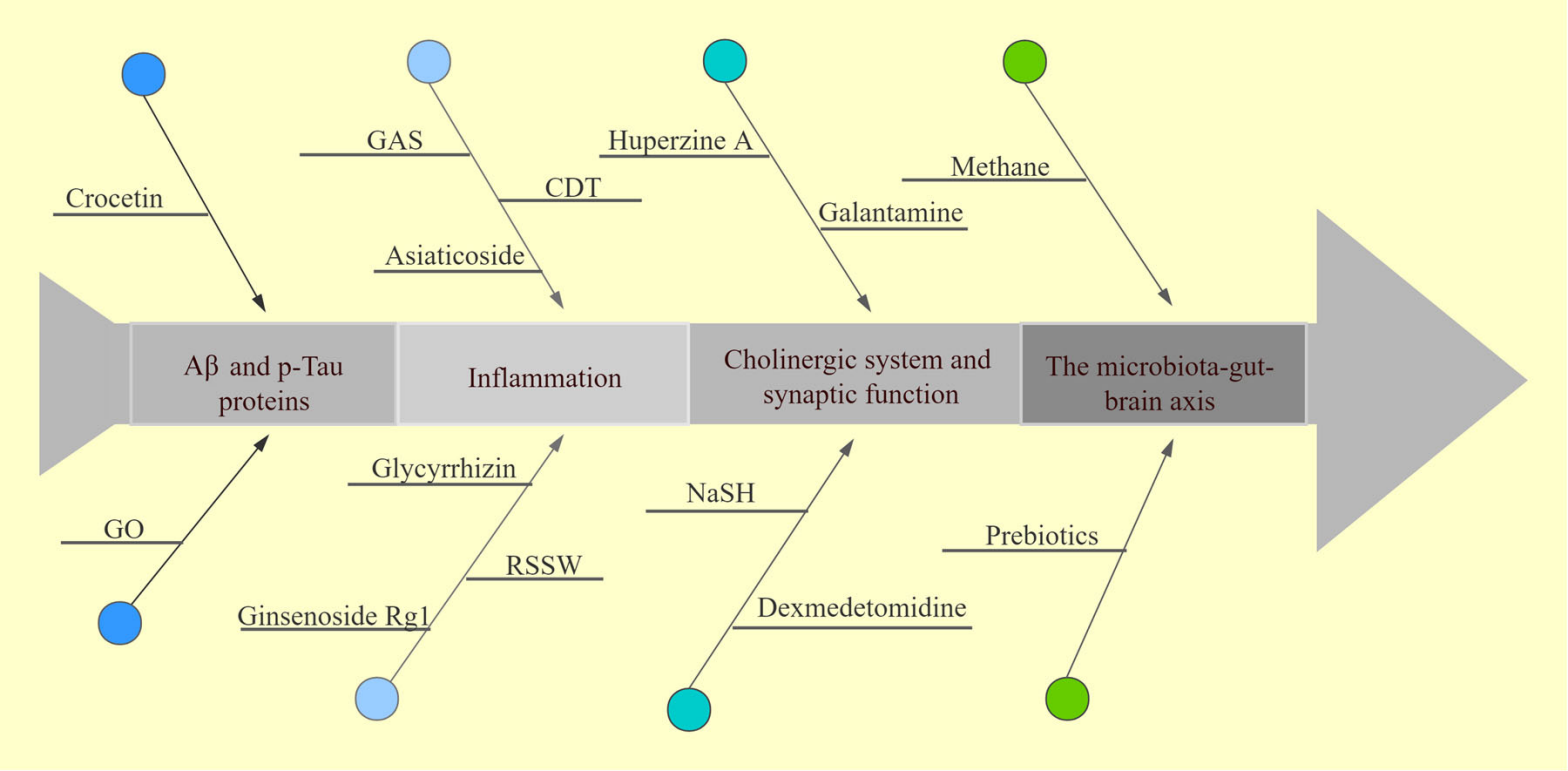
Postoperative cognitive dysfunction‐related therapeutic drugs in basic research. CDT, compound danshen tablet; GAS, gastrodia elata; GO, graphene oxide; RSSW, Renshen Shouwu extract; NaSH, sodium hydrogen sulfide. [Color figure can be viewed at wileyonlinelibrary.com]

With the increasing aging of the world, Alzheimer's disease (AD) patients are also increasing dramatically. A meta‐analysis shows that the incidence rate of AD is 11.08/1000 per year in Europe, and it increases with age.^1^ The prevalence of AD in China has reached 4%, and prevalences among men and women were 3% and 5%, respectively;^2^ moreover, the life expectancy is increasing, and the chance of surgery for the elderly and related risks deserve the attention of anesthesiologists. Postoperative cognitive dysfunction (POCD) is one of the common postoperative complications of the elderly, occurring in 12% of non‐cardiac surgery patients.^3^ In 2018, the International Naming Consensus Working Group recommended interpreting POCD as cognitive decline diagnosed preoperatively (neurocognitive impairment), any form of acute event (postoperative delirium), and cognitive decline occurring within 30 days of surgery (delayed neurocognitive recovery) and up to 12 months of being diagnosed (delayed neurocognitive recovery).^4^ We zeroed in on mental execution during the postoperative period; which actually involved the term POCD in this review. Symptoms of postoperative cognitive dysfunction are usually self‐limiting, a few will last for a long time, and the relationship between anesthesia, POCD, and dementia remains to be determined.^5^, ^6^ Patients may have delayed cognitive decline, and the probability of conversion to dementia in patients aged 65 or over is as high as 70%,^7^ and extended hospitalization time and lower quality of life are associated with an increase in morbidity and mortality^8^; recent surveys have found that POCD is the result of multiple factors in the perioperative period, and the specific mechanism warrants further investigation. Surprisingly, in 2018, the Alzheimer's Association reported that the annual socioeconomic cost of each patient with AD was 19144.36 US dollars, with a total cost of US $167.74 billion. It is estimated that the total annual cost will reach US $507.49 billion by 2030 and US $1.89 trillion by 2050,^9^ which has brought heavy burdens to families and society. Furthermore, at present, there is no effective treatment for AD, and the therapeutic effect on a target protein is not satisfactory. Therefore, it is urgent to explore new effective treatment strategies for AD and enhance postoperative cognitive recovery. In this review, we put forward the relationship and hypothesis between POCD and Alzheimer's disease. First, we outline the status of clinical and basic research on POCD and Alzheimer's disease and demonstrate the similarity between the two disease candidate pathways. The mechanism of AD to POCD may be associated with enhanced inflammation and oxidative stress, inducing neuronal apoptosis, synaptic dysfunction, central cholinergic damage, and accelerated tau protein phosphorylation, and we also found that the brain‐intestinal axis plays an important role in a variety of miRNA involved in the development of diseases and the multifactorial cascade reaction of these factors causes POCD (Figure 1). Then, based on these similar targets, we use a large number of literature databases to mine drugs and target mechanisms that may be beneficial to the disease (Figure 2). This review may be used as a guide to reduce the current situation and future of POCD patients' development into AD or treatment of AD.

## 2 CURRENT CLINICAL RESEARCH RELATED TO POCD AND AD

### 2.1 Symptoms of POCD and AD

Typically, POCD is manifested as consciousness disorders, reduced awakening levels, altered personality, impaired memory loss, difficulties in fine motor coordination, and impaired advanced cognitive function.^10^ AD is characterized by comprehensive dementia, such as memory impairment, aphasia, misuse, cognitive impairment, visual‐spatial skills, execution dysfunction, and personality and behavioral change. It is characterized by three stages, showing early onset of clinical symptoms, which gradually evolve into measurable memory, temporal, and spatial discrimination and comprehension decline, and eventually progress to memory loss, neurological changes, and even disrupts daily life.^11^, ^12^ Mild cognitive dysfunction (MIC) is considered to be a transitional phase between POCD and AD.^13^

### 2.2 Auxiliary inspection

#### 2.2.1 β‐Amyloid (Aβ) and p‐tau proteins

Aβ, including Aβ40 and Aβ42, a key component of intracellular senile plaque in patients with AD, and the p‐tau protein, the main protein component of neuronal fiber binding in neurons, is also a marker of the neurons of AD.^14^ Recently, a multiscience working group of European researchers used a structured, well‐organized, and comprehensive methodology based on an extensive literature search, prioritization of clinical questions, and grading of the quality of evidence to publish a number of clinical markers, such as cerebrospinal fluid (CSF) Aβ42, tau protein, and phosphorylated tau protein, which can identify mild cognitive impairment or AD.^15^ However, the association of these markers with POCD is still not clearly defined. A clinical study collected CSF samples from 59 patients aged 60 and older who underwent spinal and general anesthesia for elective total hip replacement, both preoperative and postoperative neuropsychological tests, and used reliable change indexes for cognitive decline at 3 and 12 months; CSF Aβ42, total tau protein, phosphorylated tau protein and neurofilament light, and compared baseline levels of biomarkers in preoperative and postoperative CSF. The results showed that the incidence of POCD after 3 months was 8.8%. Data analysis showed that low levels of Aβ42 in CSF were associated with the occurrence of POCD, which leads to cortical Aβ deposition; the increase of tau level was not associated with the susceptibility to POCD in this study,^16^ another procedure in the same clinical study, the investigator collected preoperative Aβ40/tau or Aβ42/tau ratios, a series of neuropsychology are used to judge cognitive abilities, and it was found that the cerebrospinal low Aβ40/tau ratio could specifically represent the visual memory domain, while the Aβ42/tau ratio is related to postoperative spatial perception and orientation.^17^ Regrettably, this study did not set a control group for the nonoperative group, so we could not think that this positive change will affect POCD. In another group of patients undergoing laparoscopic surgery, the plasma Aβ protein increased significantly after 24 h,^18^ on the contrary, a cardiac surgery patient was tested by PET for the overall Aβ load at 6 weeks and 1 year and received cognitive function assessment in 6 weeks and 1 year and 3 years, these results found no correlation them.^19^ This challenges the hypothesis of POCD with amyloid deposition rates. Perhaps protein accumulation is transient and coincides with postoperative cognitive dysfunction, and these relevant mechanisms need to warrant further investigation.

#### 2.2.2 Indicators of inflammatory and oxidative stress

S100beta protein is associated with neuroinflammation and dietary regulation, early low expression can protect the brain in AD patients from Aβ injury, and later increase promotes neuronal damage.^20^ Interestingly, elevated S100beta protein was found in patients with decreased cognitive function after hip replacement, and reduced expression improved cognitive function after the intervention.^21^ Interleukin (IL)‐1 can induce epileptiform activity in the brain, causing neuronal death and accelerating neurodegeneration.^22^ To verify whether serum cytokines are a stage marker between aging and AD, under strict inclusion and exclusion criteria for rigorous neuropsychological, functional, and neuropsychiatric assessments. Researchers included healthy individuals, MIC, and AD patients all in a double‐blind experiment to quantify cytokines and IL‐10 (anti‐inflammatory) in blood and CSF; and the expression of Aβ42 protein, total tau protein, and phosphorylated tau protein, hippocampus volume, and default mode network functional connectivity were carefully assessed. The results showed that mild cognitive impairment and AD increased proinflammatory cytokines, decreased the default network functional connection, increased phosphorylated tau protein and decreased Aβ protein, hippocampal volume atrophy in these patients; the anti‐inflammatory cytokine positive test showed a more favorable biomarker spectrum.^23^ However, other studies have observed that some inflammatory molecules, such as IL‐12, IL‐16, and IL‐18 in patients with severe AD are downregulated,^24^ which indicates that these inflammatory indicators have disease‐stage dependence and play dual effects, and may be related to the decreased immune response in later patients. In a clinical randomized controlled trial, the researchers found a significant increase in serum TNF‐α, IL‐1β, IL‐6, and Aβ levels in older patients on the first day of surgery, and thereafter gradually reduced.^25^ Thus, reducing Aβ expression and regulating the associated neuroinflammation of surgical traumatic stress may be a key link in the prevention of postoperative cognitive dysfunction. A recent meta‐analysis studied data available on oxidative stress and antioxidant markers in peripheral blood in patients with AD and mild cognitive impairment and found increased lipid peroxidation, copper metabolism disorders, and decreased antioxidant uric acid, vitamin A, E, C, α‐carotene, and β‐carotene, as well as total antioxidant capacity.^26^ Oxidative stress was negatively correlated with cognitive function.^27^

Is there a relationship between surgical anesthesia and AD? Neurosurgical operations have some features that are distinct from other operative procedures. There is a barrier between the blood‐brain, which has a value regarding brain function.^28^ This barrier may be disrupted in neurosurgical operations.^29^ A prospective cohort study found a higher incidence of delirium in patients undergoing craniotomy and that collapse of the blood‐brain barrier (BBB) is an early biomarker of cognitive impairment and is strongly associated with the classical susceptibility gene (the epsilon4 allele of the apolipoprotein E (APOE4)) in AD.^30^, ^31^, ^32^ The results showed that the increased pattern of total tau protein and phosphorylated tau protein in CSF was consistent with AD, and the increase of IL‐6 was correlated with inhalation anesthesia.^33^ This may increase the potential risk of postoperative cognitive decline, which is worthy of comprehensive consideration by anesthesiologists.

Recently, it has been reported that the level of peripheral acetylcholinesterase (AChE) increases in AD patients with brain Aβ protein deposition, which is synchronously associated with central AChE action.^34^ The correlation between preoperative serum anticholinergic activity (SAA) and postoperative cognitive function has been controversial. A meta‐analysis collected credible data from randomized controlled trials, longitudinal cohort studies, cross‐sectional studies, and prospective cohort studies. The results demonstrated that there were limitations in the correlation between SAA and POCD, especially in the population with basic diseases and drug treatment,^35^ and other markers need to be further found.

#### 2.2.3 Imaging representation

Harvard Medical School conducted clinical neuroimaging research^36^ and involved 1422 surgical patients. It was found that brain atrophy had no consistent correlation with POD and POCD; a further cohort study was conducted on aging patients after elective surgery. There was a relationship between preoperative diffusion MRI and POCD. The high signal and microstructure abnormalities of brain white matter shown by examination would affect the damage of network domain related to cognitive behavior, and then increase the incidence of POCD. Another study indicated that compared with normal people, the atrophy rate of the cortex, gray matter, and hippocampus increased 5–9 months after the operation, the lateral ventricle increased, and the incidence of cognitive decline increased after the operation.^13^ The decrease of structural connectivity of brain function assisted by hippocampal atrophy can be detected by MRI in AD.^37^ Older spots and nerve fiber tangles in AD patients require autopsy for detection, whereas positron emission tomography (PET) or single‐photon emission computed tomography (SPECT), an emerging technology, can detect the imaging changes, which can make the early detection of lesions a reality.^38^ PET‐analysis showed slight hemodynamic changes and abnormal cerebral metabolic rate in the decline of postoperative cognitive function.^39^

### 2.3 Diagnosis

AD mainly depends on clinical simple mental state examination (MMSE), daily life activities (ADCS‐ADL) joint research, and clinical dementia rating (CDR) of cognitive function in basic activities and tools, daily life skills, to evaluate dementia severity,^40^ others include Hopkins language learning test, Hopkins language learning comprehensive recall, brief visuospatial memory test comprehensive recall, brief visuospatial memory test delayed recall, brief visuospatial memory test comprehensive learning, brief visuospatial memory test retention Benton judgment of line orientation (JLO), and trail making B (Trails B) are also used.^17^ All that matters is to combine with the related history, biomarkers, and imaging findings and rule out other confusion caused by brain disease. The diagnosis of POCD mainly depends on the mental and psychological evaluation scale of various cognitive functions (Table 1), the subjective complaints of patients and their families, and the independence of interfering with activities of daily living are indispensable evaluation indexes. One serious issue that is all the attention keeps focused on POCD is how to deduce and calculate the details of the structure, rather than recognizing the overall concept of subtle cognitive decline. The abnormal changes in cognitive trajectory before and after anesthesia/surgery are very important, but there is no clear cause and unified diagnostic criteria for POCD, mini‐mental state examination, and montreal cognitive assessment are more commonly used.

**Table 1:** Methods of assessing postoperative cognitive dysfunction

Test	Major neuropsychological domain
Mini‐mental state examination (MMSE)	Language, calculation, orientation, memory, and so on.
Montreal cognitive assessment (MoCA)	Language, calculation, orientation, memory, executive, and so on.
Trail making test (TMT)	Executive, calculation, orientation, attention, and so on.
Weth abult intelligence scale (WAIS)	Language, executive
Wechsler memory scale (WMS)	Memory
Stroop color word test (CWT)	Memory, attention
Clock drawing test (CDT)	Comprehension, memory, executive, attention, and so on.
Loewenstein occupational therapy cognitive assessment (LOTCA)	Orientation, psychomotor, executive
Clinic dementia rating scale (CDR)	Memory, orientation, social communication ability, self‐care ability, and so on.
Alzheimer's disease assessment scale‐cognitive section (ADAS‐Cog)	Language, memory, orientation and attention, and so on.
Mini‐mental state examination	Language, memory, orientation, attention, and so on.
Cogstate software	Memory, orientation, and so on.

### 2.4 Currently commonly used clinical treatment

AChE inhibitors (rivastigmine, galantamine, and donepezil) and *N*‐methyl‐d‐aspartate (NMDA) receptor antagonists such as memantine are the mainstream drugs approved by the FDA for the treatment of AD;^17^ clinically, it cooperates with other metabolic enhancers, but these drugs are only symptomatic treatment and do not target key sites. In addition, the nature, availability, and toxic effects of drugs greatly limit their effectiveness and treatment failures often occur in clinics. At present, there is no specific treatment for patients with POCD; cognitive training has been proved to be effective,^41^ prevention strategies are mainly focused on the combination of preoperative preparation, intraoperative management, and postoperative nursing.^42^

## 3 BRIEF OVERVIEW OF BASIC PROGRESS RELATED TO AD AND POCD

### 3.1 Animal model establishment

Aging is the major risk factor for POCD.^43^ Most senescence genes are highly expressed in AD.^44^ The aging model is also one of the most widely used models in postoperative cognition. The natural aging model takes a long time and costs a lot, so the accelerated aging model is more popular. Continuous subcutaneous injection of d‐galactose into the head and neck can successfully induce the aging model of rats,^45^ and these animals show a cognitive decline and neurodegeneration.^46^ In the process of aging, cerebral blood flow generally decreases with chronic inflammation. Therefore, some scholars propose to study POCD with the rat chronic cerebral hypoperfusion model (CCH), and this model induces microglia activation and axonal injury in some brain regions (such as the caudate nucleus, optic nerve bundle, corpus callosum, and cerebral cortex) without neuronal injury.^43^ It is also a mature human vascular dementia model.^47^

### 3.2 Relevant mechanisms

#### 3.2.1 Aβ produces and removes balance destruction and tau protein phosphorylation

The amyloid hypothesis of AD is a generally accepted pathogenesis. Specifically, amyloid precursor protein (APP) exists in the cell membrane of neurons, and its terminal spans both intracellular and extracellular. Normally, it can stimulate the differentiation growth and damage of immature neurons to repair,^48^ and this process involves the cleavage of α‐secretory enzymes and γ‐secretory enzymes, and the resulting polypeptide is soluble; however, if amyloid precursor proteins are unfortunately cleaved by γ‐secretory enzymes and β‐secretory enzyme, the consequence is the production of insoluble Aβ fibrils which aggregate and spread into synaptic space, interfering with synaptic signals, impairing memory, and depositing in blood vessels also increasing the risk of haemorrhage, this abnormal polymerization will also start the pathway in neurons and activate kinases, resulting in the hyperphosphorylation of microtubule related tau proteins, mutual aggregation and entanglement, the destruction of the intracellular cytoskeleton, and the forced interruption of nutrition and signal transmission, which may eventually lead to cell apoptosis and death. These reactions can also trigger an immune response and further damage the cerebral cortex.^49^

The exact pathophysiology of POCD remains elusive, neuroinflammation and neurodegeneration are more underlying and reliable mechanisms, and surgery and/or general anesthesia may induce β amyloid accumulation and/or tau protein phosphorylation.^43^ Experiments have shown that mice undergo abdominal surgery under local anesthesia. Then, the learning and spatial memory abilities of mice were evaluated by fear test and water maze test, an Aβ42 protein and tau protein, phosphorylated protein were also examined simultaneously. The results illustrated that the severity of brain cognitive dysfunction of 18‐month‐old wild‐type and 9‐month‐old AD transgenic mice was consistent with Aβ level. Surprisingly, this did not occur in wild‐type mice for 9 months; γ‐secret inhibitor compound E improves this terrible outcome in 18‐month‐old mice. These data demonstrate that POCD may not rely on general anesthesia, and co‐collaboration with age or Aβ accumulation may be necessary for POCD, inhibiting or reducing Aβ, so the generation and deposition of Aβ is probably the key point to the efficient prevention and control of POCD.^50^

It has been found that rapamycin can inhibit the overactivation of the mTOR/p70S6K signaling pathway to downregulate Aβ42 deposition and tau protein phosphorylation.^51^ Unfortunately, the role of trauma associated with mTOR autophagy was not reflected in this study. Wenlong Li and his team researched the environmental factors that could interact with the APOE4 gene to induce the mTOR‐related pathway, depress autophagy, and activate ad‐like changes in the hippocampus.^52^ Furthermore, the imbalance of autophagy remains one of the most common causes of POCD.^53^

Exposing wild‐type and knockout homozygous adenosine A1 receptor (A1AR) mice to isoflurane, and the baseline changes of NMDA receptor subtype (NR2B), Aβ protein, and phosphorylated tau protein levels before and after anesthesia were observed, the results showed that the levels of Aβ protein and phosphorylated tau protein in gene knockout mice decreased, while those in wild‐type mice increased, and wild type mice showed worse cognitive impairment. This may be related to the activation of the adenosine A1 receptor.^54^ Triggering receptor expressed on myeloid cells 2 (TREM2) is a specific receptor for microglia,^55^ which can induce the conversion of M1 into a protective M2 phenotype that inhibits inflammatory responses.^56^ Recently, it was reported that APPswe/ps1dE9 transgenic mice were subjected to hepatectomy. The results showed that the expression of TREM2 decreased after surgery, accompanied by neuroinflammation, tau hyperphosphorylation, and cognitive disorder; adenovirus as a tool to overexpress TREM2 in AD transgenic mice resulted in the detection of high expression of microglia M2 phenotypic marker Arg1 and synaptophysin DAP12 as well as decreased expression of proinflammatory cytokine (IL‐1β), glycogen synthase kinase‐3β (GSK‐3β) and phosphorylated tau protein. The researchers concluded that high expression of TREM2 inhibits GSK‐3β Kinase activity and reduces tau protein phosphorylation to increase synaptophysin expression, inhibits the release of inflammatory factors, reduces neurotoxicity, and improves postoperative cognitive function.^47^ TREM1 has been associated with amyloid deposition in AD, and downregulated expression ameliorates memory deficits associated with impaired lipid metabolism.^57^, ^58^ This evidence suggests that a transition in the balance of TREM1/TREM2 may be associated with maintaining homeostasis in the internal brain environment, with the exact mechanism to be delved into. In contrast, TREM1 mediates neuroinflammation during an ischemic stroke by interacting with the spleen tyrosine kinase (SYK) signaling pathway and activating downstream nuclear factor kappaB (NF‐κB) and NLRP3 inflammasome.^59^

The APOE protein expressed by the APOE4 has low potency to decompose Aβ and is more prone to plaque.^60^ Studies have shown that wild‐type APOE4 mice underwent open surgery under propofol general anesthesia, and there was neuronal apoptosis in the hippocampus with CA3 Aβ deposition and hippocampal‐dependent memory impairment.^61^ The presenilin‐1 (PSEN1) and presenilin‐2 (PSEN2) gene mutations express the enzymes constituting the γ‐secretase subunit, which cleaves the APP abnormally and produces Aβ aggregation.^62^ The expression level of PSEN2 in the cortex and hippocampus of rats with cognitive dysfunction is increased,^63^ it is also essential for the learning and memory function of synapses,^64^ which may be the key points of the connection between the PSEN and POCD. Isoflurane can induce postoperative cognitive impairment, while after BACE1 gene silencing, Aβ protein production is reduced; the mechanism may be associated with the activation of PI3K/Akt signaling pathway and assembly and depression of assembly and synthesis of Aβ protein.^65^ Does anesthesia or surgery pass the mechanism of AD, which leads to POCD or aggravates AD? More research data are needed to support it.

#### 3.2.2 Oxidative stress and inflammatory response

Activated microglia is one of the early signs of AD mechanisms.^66^ Surgery and anesthesia can lead to the imbalance of neuroinflammatory homeostasis and initiate the cascade activation of cytokines and nutritional factors, such as the imbalance of the BDNF/TrkB signaling pathway, apoptosis, and reduction of dendritic spines, increasing the risk of POCD.^67^, ^68^, ^69^ Inflammation and light powder‐like protein deposition interact with each other. It has been found that soluble Aβ binds to the surface receptors of microglia to activate toxically (M1) phenotypic transformation, leading to activation of inflammation and chemokines, such as TNF‐α, IL‐6, and IL‐1β, which continue to couple the complement system to produce damaging substances such as reactive oxygen species, causing inflammation in the central nervous system. Eventually, destroying the synaptic transmission signals of hippocampal neurons in aged rats and develop into POCD.^70^

The toll‐like receptor (TLR) signaling pathway is one of the key factors in regulating the inflammatory response, but it has been reported to clean Aβ deposits in the brain;^71^ further explorations found that the aggregated Aβ is a TLR4 ligand. Long‐term contact between activated microglia and Aβ deposits will desensitize the receptor and impair the signal transduction function, leading to the disturbance of Aβ clearance and the acceleration of disease progression.^72^ And inflammation may be an early event, where small collocates have been activated before fiber binding forms and affect the homeostasis of the tau protein through a bystander effect.^73^ MAPK‐p38 overactivation is involved in the process.^74^ It is not difficult to conclude that there is a vicious circle between cytokines and amyloid deposition, which amplifies metabolic disorders and neurotoxicity, leading to neuronal dysfunction and deterioration of cognitive function. Interestingly, peripheral inflammatory factors are the bridge between the immune system and brain signal transduction, which can cross the BBB through neural and humoral pathways and activate central immune cells (such as microglia and astrocytes), affecting relevant brain areas such as the hippocampus or cortex, leading to changes in cognitive function and behaviour.^75^, ^76^ This has been proved by several studies that microarray analysis and gene ontology enrichment analysis show changes in the expression of proteins related to Ca^2+^ homeostasis in lipopolysaccharide‐induced systemic inflammation in mice, especially in AD mice;^77^ this is closely related to oxidative stress, inflammation, and apoptosis. Proinflammatory factors secreted by intestinal flora can also enter the central nervous system.^78^ TREM2 expressed on myeloid cells is expressed high in patients with AD and somewhat restricts the inflammatory response; interestingly, this effect can be suppressed by small glial TLR4, and the imbalance of TLR4/TREM2 may be the potential mechanism for AD‐related inflammation.^79^ TREM2 is also a potential target for the treatment of AD, and is this mechanism present in postoperative cognitive defects? Some research may answer this doubt for us, Jiang et al. made mice overexpress TREM2 through lentiviral infection and observed that after hepatectomy, the level of serum IL‐1β in mice was reduced and their behavior was improved.^47^ In contrast, TREM1 mediates neuroinflammation in ischemic stroke by interacting with the SYK signaling pathway to activate downstream NF‐κB and NLRP3 inflammasome.^59^

NLRP1 and NLRP3 inflammasomes mediate microglia activation and release large amounts of proinflammatory cytokines IL‐1β And IL‐18, which are involved in the occurrence of AD.^80^ IL‐1β mediates the decrease of phagocytic activity.^81^ It stimulates hyperphosphorylation of tau protein and neurofibrillary tangles,^82^ reduces the level of BDNF protein and BDNF dependent signal pathway in hippocampal synapses, thus affecting synaptic communication function,^83^ and inhibits the long‐term enhancement of the hippocampal body.^84^ These effects influence the process of learning and memory. NF‐κB has binding sites in the promoter region of genes related to amyloidosis and inflammation, which is regulated by the brain when the defense response is initiated.^85^ Interestingly, NF‐κB can upregulate the NLRP3 inflammatory body response.^86^ Sevoflurane can upregulate the expression of NF‐κB, causing cognitive dysfunction.^87^ Anesthetics may indirectly activate NLRP3 inflammatory bodies and cause a series of downstream factor changes. The role of TLR4 in the occurrence and development of POCD has been reported.^88^ Both in vivo and in vitro experiments showed that isoflurane could activate the NLRP3/caspase‐1 pathway and upregulate the secretion of IL‐18 and IL‐1β, and accelerate cognitive impairment.^89^ These studies suggest that inhibition of  overactivation of the TLR4/NF‐κB/NLRP3 pathway may be an effective strategy for POCD.

With the updation and iteration of the knowledge system, some scholars have recently put forward a hypothesis of immune vessels, which holds that aging individuals with fragile BBB and neurovascular units are more likely to have postoperative cognitive defects. Microglia and astrocytes are sensitive to molecular models related to surgical trauma.^90^

#### 3.2.3 Cholinergic receptors and synaptic plasticity

Acetylcholine modulates neuronal proliferation, differentiation, and synaptic plasticity, and enhances input processing in attention and memory contexts through signaling interaction with the prefrontal‐hippocampal network.^91^ The potential link between POCD and AD seems to depend on the impact of surgery/anesthesia on AD biomarkers and the cholinergic system. The memory decline caused by anesthesia/surgery is related to the degeneration of cholinergic neurons in the basal forebrain.^92^ In vitro and in vivo experiments show that secreted Aβ protein‐induced overactivation by the latter inhibited cholinergic function by enhancing the expression and activity of AChE.^93^ Sevoflurane can activate specific protein 1 (SP1), which leads to the inactivation of the cholinergic anti‐inflammatory pathway. Knockout of SP1 can reduce cascading inflammation and apoptosis or programmed cell death.^94^ Transgenic AD APP23 mice were more tolerant to isoflurane anesthesia.^95^

PSEN1 and PSEN2 gene mutations affect Ca^2+^ and amyloid homeostasis in AD.^62^ Brain‐derived neurotrophic factor (BDNF) can activate its full‐length receptor (TrkB‐FL), promote neuronal survival, and enhance synaptic plasticity, which is highly essential for information transmission, learning, and memory;^96^, ^97^ nevertheless, Aβ can drive the loss of BDNF function.^64^ *N*‐methyl‐d‐aspartate receptor (NMDAR) regulates the release of Ca^2+^. Strong stress induced by anesthesia and surgery accelerates nicotinamide adenine dinucleotide phosphate (NADPH) oxidase subtype NOX2 to induce microglia to release IL‐1β and downregulate BDNF, which is closely related to cognitive impairment.^98^ However, the relationship between NMDAR and BDNF is unclear. Recently, some scholars have investigated the correlation between them. The results show that anesthesia and surgery‐induced neuroinflammation can over‐activate NMDAR/Ca^2+^/calpain, then trigger the formation of truncated subtype (TrkB‐t) and intracellular domain (ICD) fragments, and lead to the imbalance of BDNF/TrkB signal pathway, trigger dendritic spine loss and apoptosis, resulting in cognitive and behavioral defects in aging rats 1–8 days after the operation.^69^

#### 3.2.4 The microbiota‐gut‐brain axis

A former study showed that intraperitoneal injection of Helicobacter pylori filtrate for 7 days significantly increased hippocampus and cortex Aβ42 deposition and the expression level of PSEN2, and a decrease in the density of mature dendritic spines in the hippocampal dentate gyrus was observed.^63^ Transfer of fecal microbiota from wild type to AD mouse model can not only improve the accumulation and aggregation of Aβ and tau proteins but also promote the recovery of neuronal dysfunction and improve cognitive dysfunction. The underlying mechanism may be related to the increase of intestinal macrophage activity and the recovery of circulating Ly6C+ monocytes.^99^ Major bacteria in the gastrointestinal tract, such as gram‐negative bacilli, *Escherichia coli* can secrete proinflammatory neurotoxins (LPS). Surprisingly, some scholars reported for the first time that bacterial lipopolysaccharide was detected in the hippocampus and cortex.^78^ Interestingly, the intestinal expression of NLRP3 was upregulated in C57BL/6 mice after transplantation of intestinal flora from AD patients, while it was the opposite in healthy people after transplantation of intestinal flora.^100^ Another study administrated probiotics to AD mice to improve the distribution of intestinal flora. The results indicated that proinflammatory cytokines IL‐1β, IL‐2, IL‐12, IFN‐γ, and TNF‐α downregulation, neuronal protease hydrolysis, and autophagy partially recovered.^101^

During the stress of operation and anesthesia, the intestinal barrier of patients was fragile. A cluster analysis of behavioral results showed that the intestinal bacteria of POCD mice changed significantly compared with non‐POCD mice, and 13 intestinal floras were significantly correlated with the results of the water maze,^102^ and researchers further indicate that isoflurane combined with tibial fracture internal fixation can be used to establish the POCD mouse model. High‐throughput 16 S rRNA sequencing was used to observe the changes of intestinal bacteria after anesthesia/surgery, and quantitative real‐time polymerase chain reaction (qRT‐PCR) was used to detect microbial strains in feces. The same changes were observed, and mixed probiotic treatment improved postoperative behavioral cognitive deficits.^103^, ^104^ However, it is still unclear whether specific bacteria play a key role. The regulation of intestinal flora is expected to become a new direction of POCD occurrence mechanism and treatment strategy in the future. Furthermore, the relationship between microorganisms and the host nervous system is complex and diverse, which is worthy of an in‐depth study by many scholars.

## 4 POTENTIAL THERAPEUTIC DRUGS FOR THE MECHANISM

### 4.1 To reduce abnormal protein deposition

The key problem to be solved in the treatment of nervous system‐related diseases is how to penetrate the BBB. Graphene oxide (GO) nanomaterials have strong penetration and show great potential in eliminating senile plaque and treating AD. Mice were subjected to bilateral intracerebral injections with GO. After treatment, the researchers received sevoflurane inhalation anesthesia combined with intramedullary fixation of lower tibial fracture. The results showed that the burden of Aβ40 and Aβ42 was reduced, and the freezing time of rats was restored partly, which improved the decline of dependent fear memory after hippocampal surgery. The mechanism is that GO binds the APP with high affinity and destroys the interaction between APP and β‐Secretase 1 (BACE1), which is related to the colocalization of BACE1 on endosomes. This laid a theoretical foundation for improving the treatment of postoperative cognitive function.^105^ Interestingly, Crocin can improve learning and memory impairment by inhibiting the activation of NF‐κB and the expression of p53 in the hippocampus of APPsw transgenic mice, reducing the deposition of inflammatory cytokines and Aβ protein, which is not mentioned in the POCD study; but based on the mechanism of AD, we can audaciously speculate that this may have the same effect in POCD.^106^

### 4.2 Reduction of neuroinflammation

Studies have shown that inhibition of the GSK‐3β pathway can upregulate IL‐10 expression;^107^ however, its mechanism in neuroprotection is uncharted. Some researchers used the virus to overexpress GSK‐3β in rats and subjected them to general anesthesia and Laparotomy, imitating POCD clinical model; the results showed that hippocampal GSK‐3β and tau protein phosphorylation increased and IL‐10 decreased. On the contrary, Gastrodin treatment can reverse this change and improve learning and memory ability 7–14 days after the operation, which shows that gastrodin can reduce GSK‐3 by downregulating GSK‐3β. Thus, it can enhance the anti‐inflammatory effect and neuroinflammation mediated by IL‐10 and inhibit the winding and aggregation of p‐tau protein.^108^ High mobility family protein 1 (HMGB1) and TLR4 binding promotes tau phosphorylation or Aβ aggregation and triggers inflammation through direct or bystander effects.^109^ Studies have shown that glycyrrhizin can anchor HMGB1, inhibit the chemotaxis and mitotic activity induced by HMGB1, and alleviate synaptic dysfunction and hippocampal neuron injury through the pathological mechanism related to AD and prevent POCD.^110^ Compared with other drugs, it has unique characteristics: high bioavailability and strong BBB penetration.^111^ For neuroinflammation caused by surgical stress, an investigation has shown that COX‐2 inhibitor parecoxib can reduce downstream inflammatory factors, including IL‐1β, IL‐6, TNF‐α, and prostaglandin E2 (PGE2) overexpression. This can protect hippocampal neurons from the stimulation of immune response and improve postoperative behavioral cognitive deficits.^112^

Asiaticoside could inhibit the apoptosis of vascular endothelial cells and restore the Aβ42‐induced decrease of mitochondrial membrane potential. The potential molecular mechanism may be downregulating the expression of TNF and IL‐6 by inhibiting the TLR4/NF‐κB signaling pathway.^113^ Other studies have shown that *Centella asiatica* prevents D‐galactose/aluminium chloride mediated neurotoxicity in rats through PP2A/GSK‐3β and apoptosis pathways.^114^ Therefore, asiaticoside is expected to be a new compound for the treatment of AD. The protective effect of dexmedetomidine‐mediated TLR4/NF‐κB signaling pathway in cognitive dysfunction has been reported.^115^ Whether *C. asiatica* can protect POCD through this pathway remains to be investigated.

Some studies have shown that ginsenoside Rg1 ameliorates lipopolysaccharide‐induced myocardial injury and inhibits inflammation, oxidative stress, and apoptosis by downregulating the TLR4/NF‐κB/NLRP3 pathway.^116^ Based on its anti‐inflammatory, antioxidant, and antiapoptotic properties, whether it has a protective effect on brain tissue under stress is the direction of our future research. Meanwhile, Renshen Shouwu extract (RSSW) could promote neurogenesis and angiogenesis after stroke through the inflammatory pathway,^117^ and dose‐dependently improve learning and memory function in rats with vascular dementia.^118^ Chicago sky blue 6B (CSB6B) may counteract Aβ‐induced cognitive impairment and neuroinflammation by inhibiting NF‐κB and NLRP3.^119^ The researchers gave 0.81 g/kg compound Danshen tablets (CDT) oral treatment to mice with cognitive impairment induced by Aβ 25–35 for 7 consecutive days. Compared with the control group, they found that CDT treatment reduced the cones in the hippocampus and CA3 area, Cell damage, IL‐6 and TNF‐α expression and average escape latency, increase ChAT (Ach biosynthetic enzyme), activated C kinase 1 (RACK1)/BDNF, increase the time spent in the target quadrant and the number of platform crossings. Therefore, CDT could balance cytokines and nutritional factors and improve cognitive defects caused by neuroinflammation.^120^ Quercetin, a natural flavonoid, was found to act as an inhibitor of microglia activation to protect against neonatal ischemic‐hypoxic encephalopathy, which coincides with the results of in vitro experiments.^58^, ^121^ Activation of microglia is a vital element in the link between neuroinflammation and POCD. The relationship between quercetin and POCD has not yet been elucidated, but we believe it is a direction worth investigating.

### 4.3 To restore cholinergic function and synaptic plasticity

Previous studies found that after surgery, AMPA receptor‐mediated EPSCs in mice were inhibited, while galantamine could enhance cholinergic tension and reduce the over‐activation of microglia in hippocampal CA1, CA3, and DG, reverse the abnormal excitatory synaptic transmission, and improve the short‐term cognitive impairment in mice.^122^  Dexmedetomidine inhibits neuroinflammation through a vagal and α7nAChR‐dependency mechanism.^123^ Donepezil had the same function on cognitive and behavioral deficits in rats. Mu‐p75‐soapwort (a cholinergic specific immunotoxin) lost its protective effect after destroying cholinergic neurons.^92^ Several lines of evidence indicated that activation of the cholinergic system has a protective response on POCD. Huperzine A is a powerful and reversible AChE inhibitor and plays an anti‐inflammatory and nutritional role.^124^ Certain chemicals such as sodium hydrogen sulfide have been found to safeguard the damage of hippocampal synaptic structure and improve spatial learning and memory function through the Warburg effect, Akt/GSK‐3β, and Notch signaling pathway.^125^, ^126^

### 4.4 Reconstruction of intestinal microflora diversity

Probiotics and prebiotics or inflammation of the intestinal flora transplantation‐mediated inhibition have the potential to become a method for the treatment of POCD.^103^, ^104^ Interestingly, one study found that dissolving methane produced by intestinal flora and injecting it into mice inhibited the NF‐κB/p38MAPK pathway, increased the expression of IL‐10 and significantly improved postperative dysfunction, and the same results were obtained in vitro experiments,^127^ IL‐10 probably is a rucial factor in reducing this immune response.

### 4.5 microRNA

miR‐200a‐3p, miR‐195, miR‐338‐5p, miR‐34a‐5p, miR‐125b‐5p, miR‐132, miR‐384, miR‐339‐5p, miR ‐135b, miR‐425‐5p, and miR‐339‐5p have been surveyed to participate in the development of AD through interaction with BACE1.^128^ The mir‐181b‐5p agomir was infused into the hippocampus and showed a downregulation of proinflammatory elements articulation and decreased microglial actuation of POCD mice.^129^ Similarly, miR‐146a was also found and is associated with the inhibition of the IRAK11/TRAF6/NF‐κB pathway and the activation of hippocampal microglia, miR‐340 is a member of the protective team.^130^, ^131^ Looking for drugs and related pathways that can regulate the expression of these tiny RNA paves the direction of future attention. In vitro, the researchers found that BV2 cells treated with dexmedetomidine could overexpress miR‐340 and improve cell viability by inhibiting the expression of NF‐κB, inflammatory cytokines TNF‐α, IL‐6, IL‐1β, IL‐2, IL‐12, and chemokine MCP‐1.^131^ Directly after, miR‐34a,^132^ miR‐381,^133^ and mi‐R129^134^ were revealed to be mediated the neuroprotective effect of dextrotomidine. miR‐410‐3p can target CXCR5 through the phosphatidylinositol 3‐kinase (PI3K)/protein kinase B (Akt) pathway to inhibit hippocampal neuron apoptosis induced by sevoflurane anesthesia.^135^

Electroacupuncture is commonly used clinically. In animal experiments, electroacupuncture could increase miR‐124 in the hypothalamus and hippocampus, and target to depress the expression of vesicle‐associated membrane protein 3(vamp3) to weaken postoperative inflammatory response.^136^ Another study suggests that miR‐124 can target Calpain small subunit 1 (Capn4) to regulate the NF‐κB signaling pathway.^137^ The targets of microRNA may be diverse and exert effects in a variety of ways.

### 4.6 Exosome effect

Interestingly, Ahmed Elsherbini and his team treated neural progenitor cells and primary cultured neurons derived from human‐induced pluripotent stem cells with exosomes isolated from the brain tissue of AD transgenic mice and the serum of AD patients. The results showed that as fission proteins increased, mitochondrial damage and apoptosis increased; this may be related to neuronal uptake and targeted transport of ceramide‐rich exosomes to mitochondria and induction of Aβ. Astrocyte‐derived exosomes mediate the binding of Aβ to voltage‐dependent anion channel 1 (VDAC1), exacerbating AD.^138^ This may be an underlying pharmacological target for the prevention of neurodegeneration.

## 5 CONCLUSIONS AND PROSPECTS

So far, there is still a controversy on the mechanism of POCD. It has been proposed that POCD may be an early stage of AD, as there are some similarities in biomakers and imaging presentations between them, implying that surgery or anaesthesia maybe a fermenting event in AD. However, basic research is still focused on animals and there is no strong clinical evidengce. It is necessary to clarify the deeper mechanism and reduce risk factors. Therefore, the study of POCD cannot be parallel to AD, even more important, it is based on AD, and contact with the anesthesia and surgical particularity of POCD, to look for the essential pathogenesis. The mechanism of POCD may additionally infiltrate AD and accelerate the progress of AD, which complement each other, and greatly improve our understanding of cognitive decline after anesthesia and surgery, especially this will transfer the research of geriatric diseases and neurodegenerative diseases to the clinical context of surgical anesthesia. Moreover, this more accurately defines POCD and strengthens the intersection with AD trajectory, combines multidisciplinary forces, and leads to new strategies to limit neuronal damage and optimize cognitive results. We need to expand into new areas of research, for example, active ingredients in traditional Chinese medicine combined with nanotechnology to better penetrate the BBB and exert efficient biological effects.

## AUTHOR CONTRIBUTIONS

Zhao‐Qiong Zhu contributed the central idea and approved this paper. Dong‐Qin Chen wrote the initial draft of the paper and completed the figure and table. Xu Fang contributed to carrying out additional analyses in this paper.

## CONFLICT OF INTEREST

The authors declare no conflicts of interest.

## ETHICS STATEMENT

Not applicable.

## Data Availability

Data sharing is not applicable to this article as no data sets were generated or analyzed during the study.
